# Trends in Serum Per- and Polyfluoroalkyl Substance (PFAS) Concentrations in Teenagers and Adults, 1999–2018 NHANES

**DOI:** 10.3390/ijerph20216984

**Published:** 2023-10-27

**Authors:** Nilisha Khadgi Sonnenberg, Akinloye Emmanuel Ojewole, Catherine Oluwalopeye Ojewole, Otite Precious Lucky, Joseph Kusi

**Affiliations:** Department of Environmental Sciences, Southern Illinois University Edwardsville, 44 Circle Drive, Campus Box 1099, Edwardsville, IL 62026, USA; nkhadgi@siue.edu (N.K.S.); emmanuelakinloye@gmail.com (A.E.O.); cojewol@siue.edu (C.O.O.); olucky@siue.edu (O.P.L.)

**Keywords:** age, per- and poly-fluoroalkyl substances, race/ethnicity, risk group, serum concentrations, sex

## Abstract

Some types of per- and poly-fluoroalkyl substances (PFAS) have been banned over the last two decades, but millions of Americans continue to have exposure to the compounds through drinking water and consumer products. Therefore, understanding the changes in serum PFAS concentrations after their limited use is necessary to protect public health. In this study, we evaluated trends of serum PFAS compounds (PFOS, PFOA, PFHxS, PFDA, and PFNA) to determine their distribution among the United States general population. We analyzed serum concentrations of PFAS measured from random subsamples of the National Health and Nutrition Examination Survey (NHANES) participants. The study results demonstrated that demographic factors such as race/ethnicity, age, and sex may influence the levels of serum PFAS over time. Adults, males, Asians, Non-Hispanic Blacks, and Non-Hispanic Whites had high risks of exposure to the selected PFAS. Overall, serum PFAS levels declined continuously in the studied population from 1999 to 2018. Among the studied population, PFOS and PFDA were the most and least prevalent PFAS in blood serum, respectively. Serum levels of PFDA, PFOA, and PFHxS showed upward trends in at least one racial/ethnic group after 2016, which underscores the need for continuous biomonitoring of PFAS levels in humans and the environment.

## 1. Introduction

In the past two decades, per- and polyfluoroalkyl substances (PFAS) have been detected around the world, including in oceans and the North Pole [1,2,3,4,5]. Global concerns about the spread of PFAS are their toxicity, bioaccumulation in the liver, and exposure in vulnerable groups. Some PFAS are persistent in the environment with extended half-lives. For instance, perfluorooctanoic acid (PFOA), perfluorooctanoic sulfonic acid (PFOS), perfluorohexane sulfonic acid (PFHxS), perfluorononanoic acid (PFNA), and perfluorodecanoic acid (PFDA) have mean half-lives of 3.8, 5.4, 8.5, 3.2, and 7.1 years, respectively [6,7], which also raises concerns about their uses and potential for human exposure over time.

PFAS are widely used in consumer products due to their chemical properties and applications in industrial processes. Most notably, PFAS have been used to make water-repellent clothing, air conditioning, non-stick cookware, carpets, cosmetics, firefighting foams, food packaging, metal plating, and semiconductors [8,9]. Additionally, PFAS are used in aerospace, automotive, electronics, and construction industries. As PFAS can resist heat, oil, grease, stains, and water, it makes the chemical befitting for consumer products [10]. Direct emissions occur during the manufacture of PFAS while indirect emissions occur at fire training grounds, landfill sites, wastewater treatment plants, airports, and military fire training areas [11]. PFAS exposure in the environment is complicated due to the nature of the sources and pathways.

Human exposure to PFAS has been linked to both occupational [6] and non-occupational settings [12]. People in communities that lack access to clean water may consume untreated water that may contain chemical pollutants [13] including PFAS. Exposure to some PFAS in the environment may cause adverse effects in humans. Studies have found an association between PFHxS in the blood serum and memory loss or confusion periods [14]. Additionally, PFDA and PFNA have been shown to cross the blood–brain barrier and have been detected in fetal cells [15]. While assessing occupational exposure is typically straightforward, the assessment of consumer exposure to PFAS can be difficult to estimate as it may take years for the symptoms to appear. Adverse health effects associated with PFAS exposure include potential endocrine toxicity, developmental deformities, carcinogenicity, and chronic cognitive toxicity [9]. The state of California has added PFOA, PFOS, and PFNA to the Proposition 65 list due to their carcinogenic and developmental effects [16]. Some studies suggest that PFAS are associated with increased risks of heart disease and cancer-related deaths among adults in the United States [17]. The toxicological mechanisms of different forms of PFAS are vastly different and may result in different health outcomes among risk groups.

Currently, the available knowledge of PFAS toxicological effects is not sufficient to understand the risk of PFAS exposures and health outcomes in minorities; therefore, further information on PFAS is critical to fill these gaps. A recent study found that Non-Hispanic Black Americans experienced higher allostatic load across various age groups [18]. Another study found that the male population of angler communities in central New York was at a significantly higher risk of PFAS exposure compared to the general population [19]. In some studies, however, low levels of PFAS have been detected in African-American communities and the predictors of the exposure included eating food prepared in nonstick cookware, consuming microwave popcorn, and dental flossing [20]. These findings suggest that lifestyles may affect the concentrations of PFAS found in blood serum of different populations. Additionally, life-long exposure among women has been associated with placental dysfunction [21]. Evidence from previous studies suggests that exposure to PFAS occurs even after certain PFAS compounds were banned, and exposure levels may vary among different groups [18,19,20,21]. The current study examined serum PFAS concentrations to determine trends and differences among demographics (races/ethnicity, gender, and age) over nearly two decades. This study would provide valuable insights into serum PFAS distribution in different populations.

## 2. Materials and Methods

### 2.1. Cohort Study

PFAS biomonitoring data used for this study were obtained from the Centers for Disease Control and Prevention (CDC) National Report on Human Exposure to Environmental Chemicals [22]. We extracted data for five PFAS, including perfluorodecanoic acid (PFDA), perfluorononanoic acid (PFNA), perfluorohexane sulfonic acid (PFHxS), perfluorooctanoic acid (PFOA), and perfluorooctanoic sulfonic acid (PFOS), concentrations detected in blood serum from subsamples of the National Health and Nutrition Examination Survey (NHANES). The CDC collected blood serum samples from participants across the country for two-year cycles from 1999 to 2018. The NHANES selects random participants that serve as a representative sample of the U.S. population based on age, gender, and race and collects data through interviews, physical examinations, and clinical analysis of serum samples.

### 2.2. Data Analysis

PFAS concentrations in blood serum samples were measured by the CDC’s Environmental Health Laboratory using mass spectrometry. Laboratory quality control and quality assurance were followed to obtain accurate measurements. The CDC measured serum levels of twelve PFAS, but we selected PFDA, PFNA, PFHxS, PFOA, and PFOS only for our study due to data availability because a higher proportion of the measurements for other PFAS was below the limit of detection. Available data for linear and branched isomers of PFOA and PFOS-n-perfluorooctanoic acid (n-PFOA), n-perfluorooctane sulfonic acid (n-PFOS), and branched perfluoromethylheptane sulfonic acid (Sm-PFOS) were also included in the study. According to the CDC Fourth National Report, isomers of PFOA and PFOS that were below the limit of detection were replaced with imputed values, and geometric means were computed using sums with at least 60% detectable values.

For this study, serum samples collected by the CDC from 18,100 participants aged ≥12 years were analyzed. The geometric means of PFAS concentrations were derived from NHANES data accounting for sample weights, stratification, and clustering survey design to obtain nationally representative estimates that could be generalized to the U.S. population. Weighted geometric means of PFDA, PFNA, PFHxS, PFOA, and PFOS for the total population, age, gender, and race/ethnicity were used to determine trends of PFAS exposure and vulnerable groups in the U.S. population. The effect of extreme values on averages was minimized using geometric means because it is less sensitive to outliers. Prior to 2013, the CDC reported total PFOA and PFOS, after which geometric means were calculated as the sum of their linear and branched isomers. The current method for calculating PFOA and PFOS may affect the trends of their serum levels for subsequent survey periods compared to previous measurements. Analyses on race were performed from 2011 to 2018 only because All Hispanic and Asian racial groups were included in the survey at the beginning of the 2011–2012 cycle. We analyzed the biomonitoring data for serum concentrations of PFAS from 1999 to 2018 according to the CDC’s analytic guidelines using RStudio software 4.2.2. (RStudio, Boston, MA, USA) version. The Shapiro–Wilk test was used to determine the normality of all datasets. We analyzed data that were normally distributed using parametric tests while those that did not show normal distribution after log transformation were analyzed using nonparametric tests. We performed one-way ANOVA followed by Tukey’s multiple comparison test to determine the differences in serum concentrations of PFDA, PFNA, PFHxS, PFOA, and PFOS over time. The effect of race/ethnicity on serum concentrations of PFDA, PFNA, PFHxS, PFOA, and PFOS was examined using one-way ANOVA followed by Tukey’s test. We determined the effect of race/ethnicity on PFHxS using the Kruskal Willis test followed by the Dunnet multiple comparison test. We determined differences in serum concentrations of all PFAS among age or sex using a two-sample *t*-test. To determine associations between PFDA, PFNA, PFHxS, PFOA, and PFOS, a correlation matrix was performed.

## 3. Results

**Figure 1:**
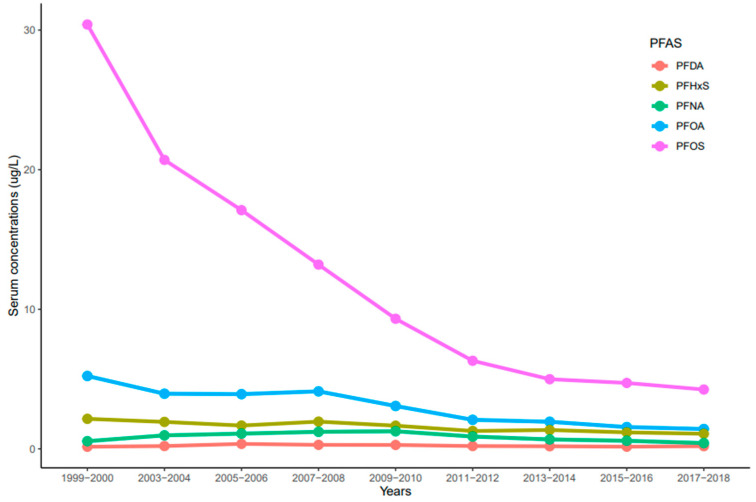
Trends of serum levels for PFDA, PFHxS, PFNA, PFOA, and PFOS among the United States population, 1999–2018.

The trends of serum PFDA, PFNA, PFHxS, PFOA, and PFOS distribution observed in NHANES participants from 1999 to 2018 reported by the CDC were influenced by time (year), race/ethnicity, age, and sex. Serum concentrations of PFDA, PFNA, PFHxS, PFOA, and PFOS ranged from 0.02 to 33.4 µg/L. In general, serum concentrations of the selected PFAS varied and decreased over time. Serum concentrations of PFOS were significantly higher than all the measured PFAS (*p* < 0.001) for all the survey periods (Figure 1). PFOS decreased rapidly between the 1999–2000 and 2011–2012 cycles and showed a continuous downward trend in 2017–2018 (Figure 1). For PFOA, its serum concentrations decreased drastically from 1999–2000 to 2003–2004, remained constant until 2007–2008, and showed a downward trend (Figure 1). PFHxS concentrations in serum decreased from 1999–2000 to 2005–2006, went up slightly during the 2007–2008 cycle, decreased again until 2011–2012, and remained constant until 2017–2018 (Figure 1). On average, PFOS, PFOA, and PFHxS serum concentrations decreased every two-year cycle by 21.3%, 14.3%, and 7.5%, respectively. Interestingly, serum concentrations of PFNA increased from 1999–2000 to 2009–2010, but concentrations decreased slowly until 2017–2018 (Figure 1). Contrary to other PFAS, PFDA concentrations in serum remained nearly constant throughout the survey periods (Figure 1). After combining the two-year cycles, concentrations of all the selected PFAS varied significantly from each other (*p* < 0.001) except PFOA and PFHxS (*p* = 0.072).

**Figure 2:**
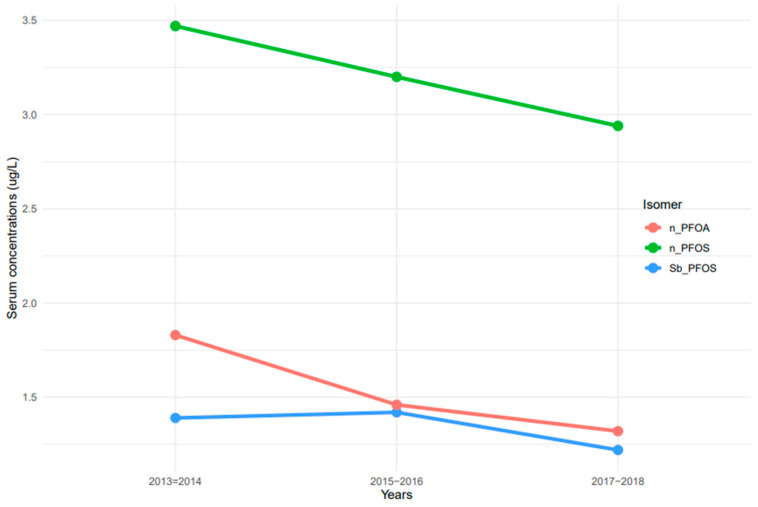
Trends of serum PFOA and PFOS linear and branched isomers among the United States population, 2013–2018. Note: n-PFOA = n-perfluorooctanoic acid, n-PFOS = n-perfluorooctane sulfonic acid, and Sm-PFOS = branched perfluoromethylheptane sulfonic acid.

The measurement of linear and branched isomers of PFOA and PFOS presented different patterns of serum concentrations. n-PFOS concentrations in serum were at least two times higher than those of n-PFOA and Sb-PFOS isomers (Figure 2). Serum concentrations of n-PFOA and n-PFOS decreased continuously from 2013–2014 to 2017–2018 (Figure 2). In contrast, serum concentrations of Sb-PFOS increased from 2013–2014 to 2015–2016, but concentrations decreased afterward.

**Figure 3:**
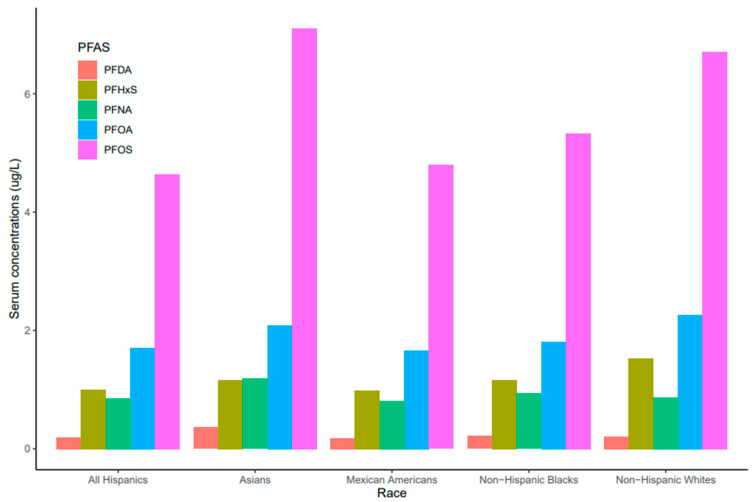
PFAS levels in serum among race/ethnicity of the United States population, 2011–2018.

**Figure 4:**
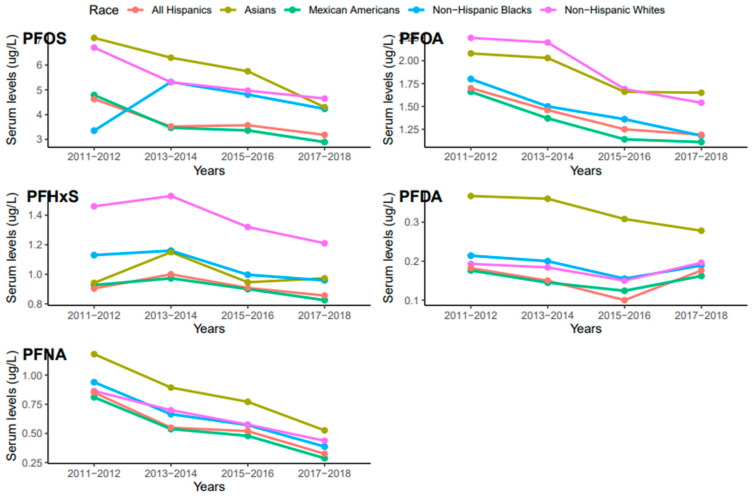
Changes in serum PFAS over time among race in the United States, 2011–2018.

Serum concentrations of PFDA, PFNA, PFHxS, PFOA, and PFO showed a common pattern across race/ethnicity where PFOS and PFNA concentrations were the highest and the lowest, respectively (Figure 3). Race/ethnicity significantly influenced serum levels for PFOA (*p* = 0.019), which were significantly higher in Non-Hispanic Whites than Mexican Americans (*p* = 0.047). PFOA concentrations in all races decreased from 2011 to 2018 but the concentration in Asians increased in 2017–2018 (Figure 4). PFOS levels in serum differed significantly among race/ethnicity (*p* = 0.009). The distribution of PFOS in serum was significantly higher in Asians than in All Hispanics and Mexican Americans (*p* < 0.05). Serum PFOS in Non-Hispanic Blacks increased rapidly by 59% from 2011–2012 to 2013–2014 and decreased by 20.5% during 2017–2018 (Figure 4). Human exposure to PFDA showed a significant difference among racial/ethnic groups (*p* < 0.001). Asians had significantly higher PFDA concentrations than All Hispanics, Mexican Americans, Non-Hispanic Blacks, and Non-Hispanic Whites (*p* < 0.01) from 2011 to 2018. PFDA concentrations in All Hispanics, Mexican Americans, Non-Hispanic Blacks, and Non-Hispanic Whites increased from 2011–2012 to 2015–2016 and showed upward trends during 2017–2018 (Figure 4). For Asians, PFDA showed a downward trend during the survey period (Figure 4). PFHxS concentrations varied significantly among the racial/ethnic groups (*p* = 0.009). Serum PFHxS in Non-Hispanic Whites was significantly higher than those of All Hispanics and Mexican Americans (*p* < 0.05). PFHxS increased from 2011 to 2012 and reached the highest concentration during 2013–2014 for all races (Figure 4). While PFHxS decreased in All Hispanics, Mexican Americans, Non-Hispanic Blacks, and Non-Hispanic Whites from 2013–2014 to 2017–2018, its concentrations in Asians increased in 2017–2018. Unlike other PFAS, serum PFNA was similar among race/ethnicity (*p* = 0.361). PFNA concentrations showed a downward trend during the survey period in all races (Figure 4).

**Figure 5:**
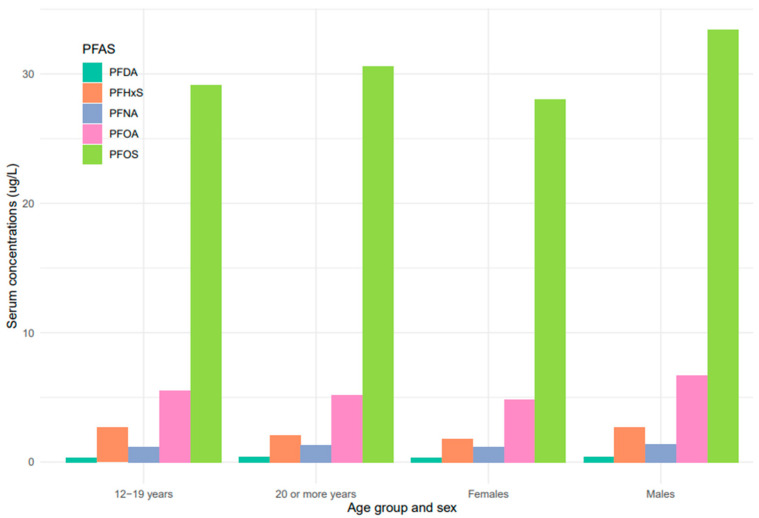
PFAS levels in serum among age groups and sex of the United States population, 1999–2018.

**Figure 6:**
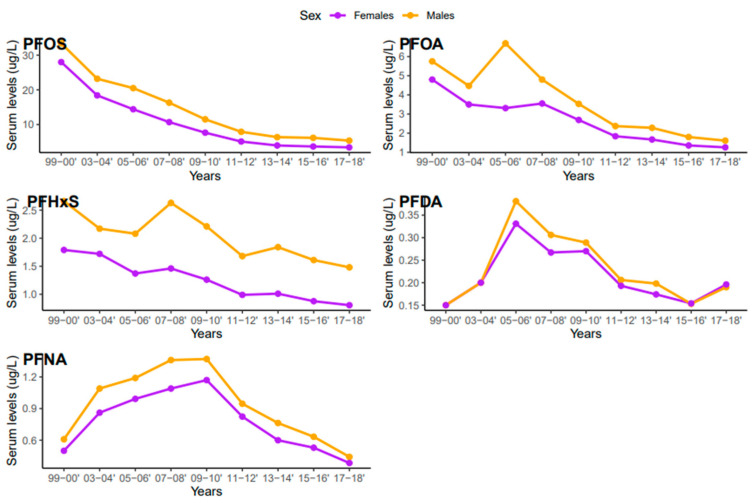
Changes in serum PFAS over time among females and males in the United States, 1999–2018.

**Figure 7:**
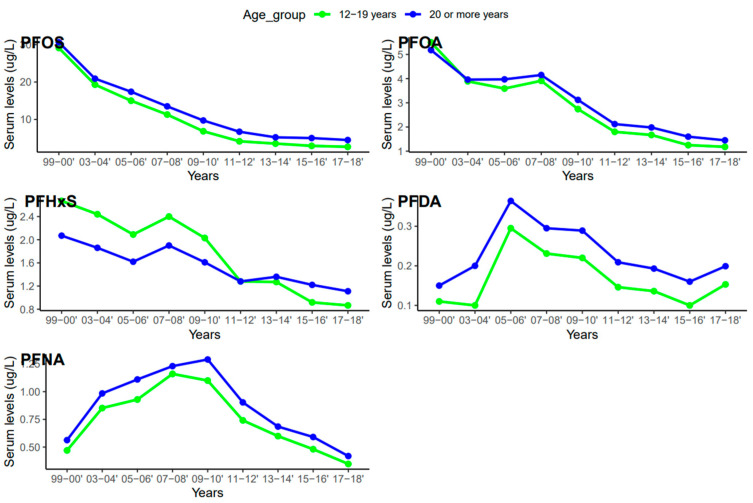
Changes in serum PFAS over time among age groups in the United States, 1999–2018.

Serum concentrations of PFDA, PFNA, PFHxS, PFOA, and PFOS differed among females and males (Figure 5). Overall, men were exposed to higher levels of all PFAS compared to women, but the difference was statistically significant for PFHxS only (*p* = 0.001). PFOS in both females and males decreased continuously from 1999–2000 to 2017–2018 (Figure 6). PFOA in males was more pronounced in 2005–2006, two times higher than that of the females (Figure 6). PFHxS exposure in both females and males showed similar downward and upward trends but the increase was more pronounced in men in 2007–2008 (Figure 6). PFDA doubled in both males and females from 1999 to 2006 and increased by 24% and 27% from 2015 to 2018 (Figure 6). Serum PFNA went up from 1999–2000 to 2009–2010 by 125% in males and 134% in females and declined steeply (Figure 6). Adults had higher levels of serum PFDA, PFNA, PFOA, and PFOS than teenagers, but the differences were not statistically significant (Figure 7). Teenagers had higher serum PFHxS than adults from 1999–2000 to 2009–2010 but had the same concentration as adults during 2011–2012 and dropped below that of adults afterward (Figure 7). All the PFAS showed downward trends in both teenagers and adults during the last five study periods except PFNA, which went up in the last cycle (Figure 7).

**Figure 8:**
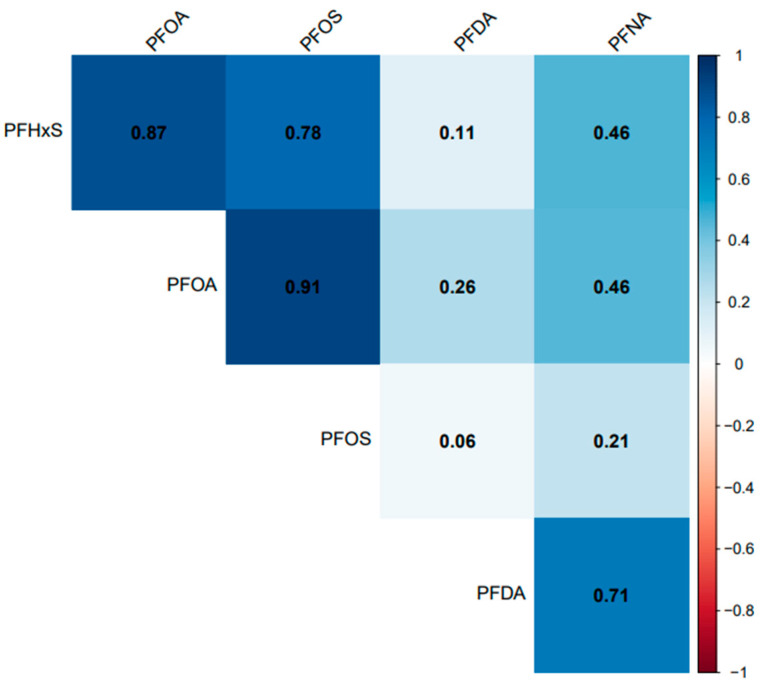
Association between serum PFDA, PFNA, PFHxS, PFOA, and PFOS in the United States, 1999–2018.

All the selected PFAS had a positive correlation with each other. PFOA, PFOS, and PFHxS had a significant (*p* = 0.001) and strong correlation (*r* > 0.77) with each other but had a weak correlation with PFDA (*r* < 0.27) (Figure 8). PFNA had a significant and strong correlation with PFDA (*r* = 0.71), the same strength of correlation with PFOA and PFHxS (*r* = 0.46), and a non-significant (*p* = 0.07) and weak correlation with PFOS (*r* = 0.21).

## 4. Discussion

### 4.1. Variations in Serum Levels of PFAS

The selected CDC’s biomonitoring data for PFAS showed downward trends and demographic variability of serum PFOS, PFOA, PFHxS, PFDA, and PFNA levels in the studied population from 1999 to 2018. The continuous decline in serum PFAS may be attributed to the ban on some PFAS being used and measures implemented by public health officials to reduce human exposure in the U.S. Although PFOS has been banned in the U.S. and other developed countries, its serum concentration in our study was significantly the highest among the selected PFAS with averages ranging from 4.3 to 30.4 µg/L, indicating that the chemical is persistent in the environment. A study conducted by the Environmental Protection Agency (EPA) in New Jersey found PFOS and PFOA concentrations ranging from 4.9 to 14 ng/L and 4.5 to 39 ng/L, respectively in drinking water supply [23]. PFOS and PFOA concentration ranges reported in the EPA study and our study exceeded the current EPA’s proposed maximum contaminant levels (MCLs) of 4 ng/L for drinking water [24]. Another study in Japan also detected PFOS and PFOA in potable tap water with concentrations ranging from 0.16 to 22 ng/L and 2.3 to 84 ng/L, respectively [25]. A similar study in Austria reported higher concentrations of PFOS over PFOA, PFHxS, PFNA, and PFDA in plasma, which is consistent with our findings [26]. The detection of PFAS in drinking water, serum, and plasma samples in previous studies demonstrates that PFAS is still available in the environment at high levels and human exposure is occurring.

PFOS is highly soluble in water [27], which may explain its higher levels in drinking water and blood serum compared to other PFAS. Again, PFOS strongly binds to albumin and plasma proteins [28,29], which could enhance the accumulation of the chemical in humans. The phase-out of PFOA and PFOS in 2002 could be responsible for their decline in blood serum observed in our study (Figure 1), but the question remains as to why PFOS occurrence is extremely higher than PFOA. Moreover, with PFOA and PFOS banned, companies have shifted to other forms of PFAS such as PFHxS which could be responsible for the slight increase in its concentration in the blood serums in 2007–2008. Occupation also plays an important role in the concentration of PFAS found in blood serum. Different types of PFAS are used in many industries, which accounted for higher workplace exposures in certain workers than in the general population [30,31].

Each PFAS has different chemical properties and behavioral patterns in the blood serum, which could account for the differences in serum concentrations observed in the current trends. PFOA and PFOS have eight carbon atoms and PFHxS has six carbon atoms bound to fluorine atoms, whereas PFNA and PFDA have eight and nine carbon atoms bound to the fluorine atoms along with carboxylic acid. These differences in chemical properties can affect their biodegradation [32] and metabolism in the human body. For example, the average half-life of PFHxS is the longest (8.5 years) among the PFAS evaluated in this study because it absorbs well and excretes slowly in humans [28]. In addition, the organic carbon partition coefficient (the ratio of a chemical’s concentration absorbed per unit mass of soil to its concentration in the aqueous phase) indicates that shorter-chained PFAS have higher water solubility while long-chained PFAS are known to adsorb highly onto sediments, which may influence the bioavailability of PFAS in blood serum [33]. This implies that long-chained PFAS such as PFOS adsorb blood particles readily, which may partly contribute to their prevalence in serum.

Levels of serum PFHxS from 2011 to 2018 were nearly constant (Figure 1), and a similar trend was observed in California teachers within the same period [34]. The current trend of serum PFHxS in the studied population suggests that exposure to the chemical, its metabolism in the body, and its release into the environment have not changed significantly over the last decade. Unlike PFHxS and other PFAS, serum PFDA increased sharply from 2015 to 2018 (Figure 1), suggesting that exposure and release of the chemical into the environment have also surged. In 2016, the U.S. Food and Drug Administration (FDA) banned the use of long-chain PFAS such as PFOA, PFOS, and PFHxS [35], which may have caused a shift towards the use of PFDA as a substitute. This may also be the reason for the rise in PFDA in blood serum (Figure 1). Similarly, the persistence of blood serum levels of PFDA in this study can be explained by the shielding effects of the fluorine atoms blocking nucleophilic attacks on the carbon chain. Furthermore, the higher trends observed with PFDA could result from the stability in the C-F bond and the longer average half-life (7 years) of PFDA in humans, which may account for the limited degradation in the environment [32].

### 4.2. Serum PFAS among Race/Ethnicity

Our analyses of NHANES data revealed significant variations in PFDA, PFHxS, PFNA, PFOA, and PFOS levels measured among different races (Figure 3). PFOS was significantly higher among all the races (Figure 3). PFOS concentrations were highest among Asians, followed by Non-Hispanic Whites and Non-Hispanic Blacks. Our results agree with the findings of [36], who reported a higher proportion of linear PFOS among Non-Hispanic Black and Asian women compared to Non-Hispanic White women. On the other hand, PFOA concentrations in this study were generally lower than those reported among different races by [37], suggesting a decline in serum PFOA and PFOS over the last decade. However, serum PFOA was more pronounced in Non-Hispanic Whites, Asians, and Non-Hispanic Blacks. The differences in serum PFOS observed in our study may be associated with diet as amounts of PFOA and PFOS were found to be substantially correlated with increased ingestion of salty foods, especially popcorn [38]. The racial distribution of PFOS and PFOA observed in our study suggests a widespread of these compounds, which remain detectable in more than 95% of the general population [39].

Temporal trends in PFHxS and PFNA concentrations were observed across races/ethnicities (Figure 4). Non-Hispanic Whites had significantly higher levels of PFHxS than Asians, Non-Hispanic Blacks, and Mexican Americans. A considerably lower concentration of PFHxS in Mexican Americans compared to Non-Hispanic Blacks and Non-Hispanic Whites has been reported [40]. A previous study found PFHxS concentrations higher than those observed in 2014–2015 and 2016–2017 (Figure 4) in pregnant African American women in Atlanta Georgia, USA [41]. However, an earlier study by [42] suggested that PFHxS could be a tracer for exposure to PFAS in consumer products, and as observed in the population of African American women, the use of consumer products was significantly associated with PFAS exposure [41]. PFAS are known to co-occur. The strong association between PFHxS and other PFAS, such as PFOA, PFOS, and PFNA (Figure 8), supports the claim that PFHxS could be used to trace PFAS exposure in consumer products.

PFNA concentrations were higher in Asians than in other races. Similar studies in the United States found that Chinese women tend to have a consistently higher concentration of PFNA compared to White and Black women [43]. PFNA was also reported to be higher in Chinese and Japanese women compared to White women [38]. The differences in PFNA concentrations observed among races may reflect the variability in lifestyle, diet, physiology, use of PFAS-containing products, or a combination of two or all these factors [36]. In addition, health disparities and environmental injustice, which mostly affect minority communities by increasing the risks of chronic diseases and environmental exposures, may influence variations in serum PFAS concentrations among races [44,45].

The NHANES data revealed a sudden change in serum PFDA levels among different ethnic groups after 2016 (Figure 4). We observed that PFDA levels in All Hispanics, Mexican Americans, Non-Hispanic Blacks, and Non-Hispanic Whites went up after the use of long-chain PFAS was banned in 2016 (Figure 4). It is possible that the production and use of short-chain PFAS including PFDA increased after the ban of the long-chain PFAS, which accounted for the current rise in PFDA exposure among the ethnic groups. Serum PFOA and PFHxS also went up in Asians from 2015 to 2018. The upward trends of PFDA, PFOA, and PFHxS in different racial groups suggest that efforts by researchers and public health officials to reduce PFAS exposure may not be enough to reduce or prevent human exposure to these chemicals. Continuous monitoring of PFDA, PFOA, and PFHxS levels in blood serum and the environment may enhance our understanding of the current exposure trends and pathways of these chemicals and other PFAS.

### 4.3. Serum Concentrations of PFAS among Male and Female

Based on the results of this study, higher concentrations of PFDA, PFHxS, PFOA, PFNA, and PFOS were observed in males than females (Figure 5). These findings are consistent with studies that found higher concentrations of PFAS in males than females in China, the United States, Germany, and Australia [40,46,47,48]. PFAS exposure pathways begin at the manufacturing sites where the compounds are synthesized and incorporated into consumer products [49]. Many men work in the manufacturing industries which increases their risks of exposure to PFAS [30]. Contrary to our study findings, many studies reported higher exposure levels of PFAS in women [50]. The lower concentration of PFAS in females observed in our study may be due to menstrual bleeding or lactation which serves as an excretion pathway for PFAS [51]. The distribution of serum levels of each PFAS in both males and females during the study period followed similar patterns, except for PFOA, where serum concentrations in males increased in 2005–2006 and dropped drastically (Figure 6).

### 4.4. Serum Concentrations of PFAS among Age Groups

The serum concentrations of PFOS, PFOA, PFHxS, PFDA, and PFNA varied across age groups. In general, PFAS levels were higher in adults ≥ 20 years except for PFHxS (Figure 5). The effect of age on serum PFAS may be associated with various factors, including exposure sources, exposure duration, bioaccumulation, and elimination processes [52,53]. Adults tend to have longer exposure time than teenagers due to their old age and occupations, so it is not surprising that they have higher levels of PFAS than teenagers. PFAS compounds have different properties, uses, and behaviors, which might lead to different patterns in their concentrations in humans over time. The increase in PFDA with age may be due to its persistent nature and slow elimination from the body [53]. Therefore, accumulation over time may lead to higher concentrations of PFDA as individuals age. An increase in serum PFNA and PFOS with age may be due to their slow elimination and potential for bioaccumulation [53]. The trend of PFHxS in teenagers was unique among the selected PFAS. Serum PFHxS for teenagers was higher than that of adults from 1999 to 2012 (Figure 7), which occurred within the same period the compound was introduced in consumer products to replace PFOS and PFOA. Similarly, PFNA in both age groups increased rapidly up to the midpoint of the study period and declined steeply (Figure 7). The sudden change in serum PFNA among age groups could be attributed to the reduction in the use of the compound in consumer products. Serum PFDA among the age groups exhibited sporadic changes during the survey period. The recent increase in serum levels of PFDA in both teenagers and adults is common across races and genders (Figure 4 and Figure 6), suggesting that there may be ongoing exposure to this PFAS.

## 5. Conclusions

Serum PFAS declined and varied among demographics from 1999 to 2018 in the studied population. Although PFAS have been around for nearly a century, this study revealed a decline in serum PFOA, PFOS, PFHxS, PFNA, and PFDA in the studied population. Many studies focused more on PFOA and PFOS while other PFAS, such as PFHxS, PFNA, and PFDA, highlighted in this study, remain an equal problem. After the ban on long-chain PFAS in 2016, PFDA serum levels seem to have risen in recent years suggesting continuous exposure to the chemical. Serum PFAS differed among age groups, gender, and race/ethnicity. Adults, males, Asians, Non-Hispanic Blacks, and Non-Hispanic Whites seem to have high risks of exposure to PFAS. Developing a coordinated plan to learn more about PFAS and support the government’s mitigation actions for a complete ban on PFAS and develop substitutes might be necessary. Some of the PFAS analyzed by the CDC laboratory were below the limit of detection. It is possible that the current techniques and technologies are not able to detect some PFAS in biological and environmental samples at very low levels. Investing in advanced techniques and technologies may allow researchers to detect all kinds of PFAS in the environment. Furthermore, PFAS studies mainly focus on the physical impacts of exposure. However, more attention needs to be given to minority communities. The findings of this study may inform health professionals and policymakers to develop and implement interventions that could reduce PFAS exposure and its adverse effects on vulnerable populations.

### Limitation to the Study

Information about the exposure of individuals to different sources of PFAS whose blood sample measurements were used for the analysis was not provided at the time of carrying out this research. It should be noted that exposure to different sources may also have an impact on PFAS levels in blood serum and other human tissue.

## Data Availability

Publicly available datasets were analyzed in this study. This data can be found here: https://www.cdc.gov/exposurereport/data_tables.html (accessed on 15 July 2023).

## References

[1] Zhao Y.G., Wong C.K.C., Wong M.H. (2012). Environmental contamination, human exposure and body loadings of perfluorooctane sulfonate (PFOS), focusing on Asian countries. Chemosphere.

[2] Holmström K.E., Järnberg U., Bignert A. (2005). Temporal trends of PFOS and PFOA in guillemot eggs from the Baltic Sea, 1968–2003. Environ. Sci. Technol..

[3] Cai M., Zhao Z., Yin Z., Ahrens L., Huang P., Cai M., Yang H., He J., Sturm R., Ebinghaus R. (2012). Occurrence of perfluoroalkyl compounds in surface waters from the North Pacific to the Arctic Ocean. Environ. Sci. Technol..

[4] Bossi R., Riget F.F., Dietz R., Sonne C., Fauser P., Dam M., Vorkamp K. (2005). Preliminary screening of perfluorooctane sulfonate (PFOS) and other fluorochemicals in fish, birds and marine mammals from Greenland and the Faroe Islands. Environ. Pollut..

[5] Ghisi R., Vamerali T., Manzetti S. (2019). Accumulation of perfluorinated alkyl substances (PFAS) in agricultural plants: A review. Environ. Res..

[6] Olsen G.W., Burris J.M., Ehresman D.J., Froelich J.W., Seacat A.M., Butenhoff J.L., Zobel L.R. (2007). Half-life of serum elimination of perfluorooctanesulfonate, perfluorohexanesulfonate, and perfluorooctanoate in retired fluorochemical production workers. Environ. Health Perspect..

[7] Marlissa Campbell A.K., Li L.-H., Iyer P., Moran F., Kaufman F., Niknam Y. (2021). Proposition 65 Evidence on the Male Reproductive Toxicity of Perfluorononanoic Acid (PFNA) and Its Salts and Perfluorodecanoic Acid (PFDA) and Its Salts. https://oehha.ca.gov/media/downloads/crnr/pfnapfdahid100121.pdf.

[8] Glüge J., Scheringer M., Cousins I.T., DeWitt J.C., Goldenman G., Herzke D., Lohmann R., Ng C.A., Trier X., Wang Z. (2021). An overview of the uses of per- and polyfluoroalkyl substances (PFAS). Environ. Sci. Process. Impacts.

[9] Knutsen H.K., Alexander J., Barregård L., Bignami M., Brüschweiler B., Ceccatelli S., Cottrill B., Dinovi M., Edler L., Grasl-Kraupp B. (2018). Risk to human health related to the presence of perfluorooctane sulfonic acid and perfluorooctanoic acid in food. EFSA J..

[10] Miller A., Elliott J.E., Elliott K.H., Lee S., Cyr F. (2015). Temporal trends of perfluoroalkyl substances (PFAS) in eggs of coastal and offshore birds: Increasing PFAS levels associated with offshore bird species breeding on the Pacific coast of Canada and wintering near Asia. Environ. Toxicol. Chem..

[11] Brase R.A., Mullin E.J., Spink D.C. (2021). Legacy and emerging per-and polyfluoroalkyl substances: Analytical techniques, environmental fate, and health effects. Int. J. Mol. Sci..

[12] Trudel D., Horowitz L., Wormuth M., Scheringer M., Cousins I.T., Hungerbühler K. (2008). Estimating consumer exposure to PFOS and PFOA. Risk Anal..

[13] Kusi J., Scheuerman P.R., Maier K.J. (2020). Antimicrobial properties of silver nanoparticles may interfere with fecal indicator bacteria detection in pathogen impaired streams. Environ. Pollut..

[14] Sim K.H., Lee Y.J. (2022). Perfluorohexane sulfonate induces memory impairment and downregulation of neuroproteins via NMDA receptor-mediated PKC-ERK/AMPK signaling pathway. Chemosphere.

[15] Bell K.S., O’Shaughnessy K.L. (2022). The development and function of the brain barriers—An overlooked consideration for chemical toxicity. Front. Toxicol..

[16] Garfield J., Harris S. (2023). Chemicals Known to the State to Cause Cancer or Reproductive Toxicity.

[17] Wen X., Xu X. (2022). Exposure to Per- and Polyfluoroalkyl Substances and Mortality in U.S. Adults: A Population-Based Cohort Study. Environ. Health Perspect..

[18] Bashir T., Obeng-Gyasi E. (2022). The Association between Multiple Per-and Polyfluoroalkyl Substances’ Serum Levels and Allostatic Load. Int. J. Environ. Res. Public Health.

[19] Wattigney W.A., Savadatti S.S., Liu M., Pavuk M., Lewis-Michl E., Kannan K., Wang W., Spliethoff H., Marquez-Bravo L., Hwang S.A. (2022). Biomonitoring of per- and polyfluoroalkyl substances in minority angler communities in central New York State. Environ. Res..

[20] Boronow K.E., Brody J.G., Schaider L.A., Peaslee G.F., Havas L., Cohn B.A. (2019). Serum concentrations of PFASs and exposure-related behaviors in African American and non-Hispanic white women. J. Expo. Sci. Environ. Epidemiol..

[21] Blake B.E., Fenton S.E. (2020). Early life exposure to per- and polyfluoroalkyl substances (PFAS) and latent health outcomes: A review including the placenta as a target tissue and possible driver of peri- and postnatal effects. Toxicology.

[22] Centers for Disease and Control Prevention Biomonitoring Data Tables for Environmental Chemicals. https://www.cdc.gov/exposurereport/data_tables.html.

[23] New Jersey Department of Environmental Protection Determination of perfluorooctanoic acid (PFOA) in aqueous samples: Final report. http://hdl.handle.net/10929/25465.

[24] USEPA (2023). EPA’s Proposal to Limit PFAS in Drinking Water. https://www.epa.gov/system/files/documents/2023-04/Fact%20Sheet_PFAS_NPWDR_Final_4.4.23.pdf.

[25] Takagi S., Adachi F., Miyano K., Koizumi Y., Tanaka H., Mimura M., Watanabe I., Tanabe S., Kannan K. (2008). Perfluorooctanesulfonate and perfluorooctanoate in raw and treated tap water from Osaka, Japan. Chemosphere.

[26] Forsthuber M., Kaiser A.M., Granitzer S., Hassl I., Hengstschläger M., Stangl H., Gundacker C. (2020). Albumin is the major carrier protein for PFOS, PFOA, PFHxS, PFNA and PFDA in human plasma. Environ. Int..

[27] EPA (2016). Drinking Water Health Advisory for Perfluorooctanoic Acid (PFOA).

[28] Pizzurro D.M., Seeley M., Kerper L.E., Beck B.D. (2019). Interspecies differences in perfluoroalkyl substances (PFAS)toxicokinetics and application to health-based criteria. Regul. Toxicol. Pharmacol..

[29] Chi Q., Li Z., Huang J., Ma J., Wang X. (2018). Interactions of perfluorooctanoic acid and perfluorooctanesulfonic acid with serum albumins by native mass spectrometry, fluorescence and molecular docking. Chemosphere.

[30] Agency for Toxic Substances and Disease Registry PFAS in the U.S. Population. https://www.atsdr.cdc.gov/pfas/health-effects/us-population.html.

[31] Christensen B.T., Calkins M.M. (2023). Occupational exposure to per- and polyfluoroalkyl substances: A scope review of the literature from 1980–2021. J. Expo. Sci. Environ. Epidemiol..

[32] European Chemicals Agency (ECHA) (2016). Substance Name: Nonadecafluorodecanoic Acid (PFDA) and Its Sodium and Ammonium Salts. https://echa.europa.eu/documents/10162/77191b64-4f79-0e53-0b89-57098caf46c4.

[33] Dalahmeh S., Tirgani S., Komakech A.J., Niwagaba C.B., Ahrens L. (2018). Per- and polyfluoroalkyl substances (PFASs) in water, soil and plants in wetlands and agricultural areas in Kampala, Uganda. Sci. Total Environ..

[34] Hurley S., Goldberg D., Wang M., Park J.S., Petreas M., Bernstein L., Anton-Culver H., Nelson D.O., Reynolds P. (2018). Time Trends in Per- and Polyfluoroalkyl Substances (PFASs) in California Women: Declining Serum Levels, 2011–2015. Environ. Sci. Technol..

[35] U.S. Food and Drug Administration (2016). Authorized Uses of PFAS in Food Contact Applications. https://www.fda.gov/food/process-contaminants-food/authorized-uses-pfas-food-contact-applications#:~:text=In2016%2CtheFDArevoked,soldintheUnitedStates.

[36] Kang H., Calafat A.M., Karvonen-Gutierrez C.A., Park S.K. (2023). Isomer-Specific Serum Concentrations of Perfluorooctane Sulfonic Acid among U.S. Adults: Results from the National Health and Nutrition Examination Survey (NHANES) and the Study of Women’s Health Across the Nation Multi-Pollutant Study (SWAN-MPS). Environ. Sci. Technol..

[37] Kato K., Wong L.Y., Jia L.T., Kuklenyik Z., Calafat A.M. (2011). Trends in exposure to polyfluoroalkyl chemicals in the U.S. population: 1999–2008. Environ. Sci. Technol..

[38] Park S.K., Peng Q., Ding N., Mukherjee B., Harlow S.D. (2019). Determinants of per- and polyfluoroalkyl substances (PFAS) in midlife women: Evidence of racial/ethnic and geographic differences in PFAS exposure. Environ. Res..

[39] Calafat A.M., Kato K., Hubbard K., Jia T., Botelho J.C., Wong L.Y. (2019). Legacy and alternative per- and polyfluoroalkyl substances in the U.S. general population: Paired serum-urine data from the 2013–2014 National Health and Nutrition Examination Survey. Environ. Int..

[40] Calafat A.M., Wong L.Y., Kuklenyik Z., Reidy J.A., Needham L.L. (2007). Polyfluoroalkyl chemicals in the U.S. population: Data from the national health and nutrition examination survey (NHANES) 2003–2004 and comparisons with NHANES 1999–2000. Environ. Health Perspect..

[41] Chang C.J., Ryan P.B., Smarr M.M., Kannan K., Panuwet P., Dunlop A.L., Corwin E.J., Barr D.B. (2021). Serum per- and polyfluoroalkyl substance (PFAS) concentrations and predictors of exposure among pregnant African American women in the Atlanta area, Georgia. Environ. Res..

[42] Hu X.C., Dassuncao C., Zhang X., Grandjean P., Weihe P., Webster G.M., Nielsen F., Sunderland E.M. (2018). Can profiles of poly- and Perfluoroalkyl substances (PFASs) in human serum provide information on major exposure sources?. Environ. Health.

[43] Ding N., Harlow S.D., Batterman S., Mukherjee B., Park S.K. (2020). Longitudinal trends in perfluoroalkyl and polyfluoroalkyl substances among multiethnic midlife women from 1999 to 2011: The Study of Women′s Health Across the Nation. Environ. Int..

[44] Kramer M.R., Hogue C.R. (2009). What causes racial disparities in very preterm birth? A biosocial perspective. Epidemiol. Rev..

[45] Dunlop A.L., Salihu H.M., Freymann G.R., Smith C.K., Brann A.W. (2011). Very low birth weight births in Georgia, 1994–2005: Trends and racial disparities. Matern. Child Health J..

[46] Guo F., Zhong Y., Wang Y., Li J., Zhang J., Liu J., Zhao Y., Wu Y. (2011). Perfluorinated compounds in human blood around Bohai Sea, China. Chemosphere.

[47] Fromme H., Midasch O., Twardella D., Angerer J., Boehmer S., Liebl B. (2007). Occurrence of perfluorinated substances in an adult German population in southern Bavaria. Int. Arch. Occup. Environ. Health.

[48] Kärrman A., Mueller J.F., Van Bavel B., Harden F., Toms L.M.L., Lindström G. (2006). Levels of 12 perfluorinated chemicals in pooled Australian serum, collected 2002–2003, in relation to age, gender, and region. Environ. Sci. Technol..

[49] Sunderland E.M., Hu X., Dassuncao C. (2019). A review of pathways of human exposure to PFAS. Physiol. Behav..

[50] Kärrman A., Ericson I., VanBavel B., Ola Darnerud P., Aune M., Glynn A., Ligneli S., Lindström G. (2007). Exposure of perfluorinated chemicals through lactation: Levels of matched human milk and serum and a temporal trend, 1996–2004, in Sweden. Environ. Health Perspect..

[51] Bjerregaard-Olesen C., Bach C.C., Long M., Ghisari M., Bossi R., Bech B.H., Nohr E.A., Henriksen T.B., Olsen J., Bonefeld-Jørgensen E.C. (2016). Time trends of perfluorinated alkyl acids in serum from Danish pregnant women 2008–2013. Environ. Int..

[52] Zhang T., Wu Q., Sun H.W., Rao J., Kannan K. (2010). Perchlorate and iodide in whole blood samples from infants, children, and adults in Nanchang, China. Environ. Sci. Technol..

[53] Haug L.S., Thomsen C., Becher G. (2009). Time trends and the influence of age and gender on serum concentrations of perfluorinated compounds in archived human samples. Environ. Sci. Technol..

